# The new philosophy of psychiatry: its (recent) past, present and future: a review of the Oxford University Press series *International Perspectives in Philosophy and Psychiatry*

**DOI:** 10.1186/1747-5341-2-9

**Published:** 2007-06-08

**Authors:** Natalie F Banner, Tim Thornton

**Affiliations:** 1 Institute for Philosophy, Diversity and Mental Health, University of Central Lancashire, Preston, UK

## Abstract

There has been a recent growth in philosophy of psychiatry that draws heavily (although not exclusively) on analytic philosophy with the aim of a better understanding of psychiatry through an analysis of some of its fundamental concepts. This 'new philosophy of psychiatry' is an addition to both analytic philosophy and to the broader interpretation of mental health care. Nevertheless, it is already a flourishing philosophical field. One indication of this is the new Oxford University Press series *International Perspectives in Philosophy and Psychiatry *seven volumes of which (by Bolton and Hill; Bracken and Thomas; Fulford, Morris, Sadler, and Stanghellini; Hughes, Louw, and Sabat; Pickering; Sadler; and Stanghellini) are examined in this critical review.

## Background: the recent past

Philosophy of psychiatry in the English-speaking world has broad historical roots, ranging across many traditions of thought in Europe, the UK and the USA. The phenomenological-existential tradition of psychoanalytic theory, Anglo-American analytic philosophy and streams of the Continental tradition have all influenced and shaped the field. However, in recent years there has been a growth in philosophy of psychiatry which draws heavily (although not exclusively) on broadly analytic philosophy and which aims to at a better understanding of psychiatry through an analysis of some of its fundamental concepts.

This 'new philosophy of psychiatry' is thus a comparatively recent addition to both analytic philosophy and to the broader interpretation of mental health care. Nevertheless, it is already a flourishing philosophical field. Over the last fifteen years or so there has been considerable research activity in this area. Signs of that activity include:

• the launch of the journal *Philosophy, Psychiatry and Psychology *in 1994 co-edited in the US and UK, supported by the Association for the Advancement of Philosophy and Psychiatry (AAPP) which fosters close associations with the American Psychiatric Association.

• the two main centres of excellence in the UK: the masters programme in philosophy of mental disorder at King's College London and the new Institute for Philosophy, Diversity and Mental Health at the University of Central Lancashire which will shortly launch its own distance learning masters in Philosophy and Mental Health based on the *Oxford Textbook of Philosophy and Psychiatry*.

• the established annual series of International Conferences on Philosophy, Psychiatry and Psychology which has met recently in Florence, Heidelberg, Yale and Leiden and will be at Sun City, South Africa in 2007. Conferences are planned till 2012.

• the creation of a Special Interest Group in Philosophy within the Royal College of Psychiatrists in the UK, which currently has over 1200 members.

In addition, the new Oxford University Press book series *International Perspectives in Philosophy and Psychiatry *– which also includes the *Companion to the Philosophy of Psychiatry *[1] and the *Oxford Textbook of Philosophy and Psychiatry *[2] – has now published more than ten volumes. It seems therefore that now is a particularly appropriate time to examine the developing field as well as reviewing 7 of the IPPP titles below.

As well as its youth, the new philosophy of psychiatry has two further features that make it stand out. Firstly, it is not a 'natural kind'. By that we mean that there is not an established set of inter-related problems with familiar, if rival, solutions. Published work is, instead, generally drawn from parent sub-disciplines within philosophy such as philosophy of mind, of science and of values and ethics. It is an area where philosophical methods, accounts and theories can be applied to psychiatric phenomena and thus it also serves to test those accounts. To take one type of example, psychopathology is a test track for theories in the philosophy of mind. Symptoms such as thought insertion, where subjects experience their thoughts as somehow not their own, challenge accounts of the everyday 'ownership' of thoughts. But there is also traffic the other way. Three centuries of discussing the relationship of mind and body have furnished philosophers with a variety of subtle models (from forms of dualism, through gradations of physicalism, to eliminativism with modern alternatives such as enactivism) which can help in the interpretation of psychiatric data.

Secondly, unlike some areas of philosophy, philosophy of psychiatry can have a genuine impact on practice. It is a philosophy of, and for, mental health care. It provides tools for critical understanding of contemporary practices, and of the assumptions on which mental health care more broadly, and psychiatry more narrowly, are based. Thus it is not merely an abstract area of thought and research, of interest only to academics. In providing a deeper, clearer understanding of the concepts, principles and values inherent in everyday thinking about mental health, psychiatric diagnoses and the theoretical drivers of mental health policy, it can impact directly on the lives of people involved in all aspects of mental health care.

A brief examination of the history of the subject reveals why the discipline of psychiatry is particularly suited to contributions from philosophy. Whilst the father of psychopathology, the German philosopher and psychiatrist Karl Jaspers, combined psychiatric and philosophical expertise, within the English speaking tradition philosophy and psychiatry went their separate ways throughout most of the twentieth century. (By contrast, in mainland Europe the connection between psychiatry and phenomenological philosophy has continued since Jaspers' day.)

But towards the end of the twentieth century, the rise of the anti-psychiatry movement prompted a resurgence of philosophical interest in psychiatry. This was because a key element of the anti-psychiatric criticism of mental health care turned on a contentious claim about the nature of mental illness: mental illness does not exist; it is a myth. Such a sceptical claim is paradigmatically philosophical and one of the main proponents of anti-psychiatry, the psychiatrist Thomas Szasz, put forward a number of philosophical arguments in support of it. These turned on the fact that psychiatric diagnosis is essentially evaluative. From this he concluded that, unlike physical illness, it could not be medically treated because as illness it was not real. (The apparent reality of mental illness is best explained, according to Szasz, as the reality of non-medically treatable life problems.)

Szasz's sceptical arguments spurred responses by both psychiatrists and philosophers questioning whether diagnosis is, after all, essentially evaluative and, if it is, whether Szasz's conclusions followed. Thus the analysis of mental illness, and the role of values in that analysis, lies at the heart of recent philosophy of psychiatry.

In addition to the importance of values, two further key areas of mental health care prompt immediate philosophical questioning. Firstly, psychiatry since Jaspers has sought to balance two key elements: investigation of the bio-medical facts and empathic investigation of subjects' experiences. Both bio-medical facts and meanings (broadly construed to include experiences, beliefs and utterances) need somehow to be integrated into mental health care. This marks a sharp delineation from other areas of medicine where subjects' experiences are subordinate to the physically described symptoms and organic pathology with which they present. By contrast, psychiatric disorders seem to involve problems of the 'self' (however this is construed) in which experiences, behaviour and beliefs play a fundamentally important role in the onset, course and recovery of symptoms.

This raises questions of both the nature of the *distinction *between explanation according to the canons of the natural sciences (the 'realm of law') and understanding meaningful connections (in the 'space of reasons') and the *relationship *between natural scientific facts and meanings. If there is a clear distinction and meanings are conceptually irreducible to biomedical facts, efforts to understand the nature of this relationship become all the more philosophically interesting.

Secondly, there has been much work by psychiatrists since the Second World War to develop psychiatric classification or taxonomy. This has, historically, been in response to a concern about a lack of agreement or reliability about psychiatric diagnosis. More recently, there has been growing concern that reliability has been improved but only at the cost of validity, or underlying truth, of classificatory schemas. The worry is that psychiatric diagnostic systems may not 'carve nature at the joints'. This concern has also been reflected in philosophy of psychiatry as an instance of a broader question of the role of science in mental health care.

The *International Perspectives in Philosophy and Psychiatry *series already contains a number of different works which begin to show the wide variety contained within philosophy of psychiatry, some of which we review in more detail below. In accord with the comments above, three broad interconnected themes are already clear. They concern:

• the role of values in psychiatric diagnosis and treatment;

• the question of the place of understanding subjects' experiences, their meanings and the relationship of understanding to natural scientific explanation

• the scientific status of the 'facts' or 'evidence' that contribute towards psychiatric diagnoses

This is not to say that the books discussed below fit neatly into just one of the categories. John Sadler's *Values and Psychiatric Diagnosis*, for example, concerns the interplay of values and scientific evidence. Whilst Pat Bracken and Phil Thomas' *Postpsychiatry *focuses mainly on the third aspect, it has something to say about the other two areas as well. Nevertheless, it is useful to think of these as forming the main foci of work in this area to provide some kind of overview of a diverse field.

We will now turn to seven of the works recently published in the Oxford University Press *International Perspectives in Philosophy and Psychiatry *series.

## The present: International Perspectives in Philosophy and Psychiatry 

### John Sadler *Values and Psychiatric Diagnosis*

John Sadler's *Values and Psychiatric Diagnosis *[3] looks to be concerned with the first area of our three-part distinction: values. But, in fact, it is equally concerned with the third area: the nature of the scientific project of framing psychiatric classification. Values and classification go hand in hand for reasons that will become clear.

The book starts with two key claims. Firstly, he claims that psychiatry is thoroughly charged with values but, at the same time, it disguises or denies the role that values play. Thus one key aim of his book is to explore the multiple roles of values in a variety of different areas. These include broad themes such as the patient and professional roles, technology, culture and politics. But it also concerns more specific areas of psychiatric interest such as sex and gender and genetics.

Sadler's second key claim follows from a further assumption he makes. Because classification lies at the heart of the scientific self-image of the profession, light can be shed on psychiatry by looking at psychiatric classification in particular.

Why does this relate back to values? This stems from an initially surprising result of examining the evidence. Published expressions of the aims for, and methodological assumptions of, recent DSM and ICD classifications in their introductions and so forth reveals a striking consistency. But at the same time, the evolution of psychiatric classification over the last fifty years has been anything but gradual. This presents a tension:

We seem to have a paradox: the psychiatric nosologists seem to develop a significant degree of consensus about what they want in a diagnostic system, yet substantial changes occur over the years – even dramatic ones. How does this occur? What forces shape the changes? [4]

Of course, one response to this question might have been that empirical findings have driven the changes. But Sadler suggests that this is not the right answer which should, instead, be derived from Thomas Kuhn's analysis of science. Just as Kuhn argued that values play a key role in explaining scientific disputes so changes in classification can be best explained through disagreements about the values that play an essential role in scientific psychiatry. Thus *Values and Psychiatric Diagnosis *examines the role that values play across a variety of contexts but it takes classification to be of central importance because by looking to the values that drive it, light can be shed on psychiatry as a whole.

Admirably for work in this area, Sadler spends some time giving an overview of what he understands values to be. Following the influence of pragmatist philosophy he takes them to guide actions and also to justify praise or blame. He distinguishes between 'thick' and 'thin' value-terms, value-semantics, value-commitments, value-entailments, and value-consequences and also suggests that there is a variety of kinds of value: aesthetic, ethical, pragmatic, epistemic, and ontological. With this framework in place the bulk of the book sets about unpacking and analysing the value commitments and disagreements in a number of areas of psychiatry.

Given the broad canvas of the investigation it is perhaps unsurprising that no single line of argument emerges. But one interesting discussion arises in the chapter on sex and gender. Having outlined the recent history of diagnoses of homosexuality, gender identity disorder and paraphilia, Sadler suggests that these reveal a fundamental difficulty for psychiatry. He quotes a qualification made in the DSM-IV definition of disorder:

Neither deviant behaviour (eg. political, religious, or sexual) nor conflicts that are primarily between the individual and society are mental disorders unless the deviance or conflict is a symptom of a dysfunction in the individual. [5]

But, as Sadler argues, this raises a question of how one does define disorder.

One difficulty with this DSM rubric, and one that limits its applicability to the issue of unconventional sexual practices or gender behaviour, is how one determines whether a given behaviour or set of behaviours is dysfunctional. [6]

Having already argued that diagnosis is essentially evaluative, Sadler suggests that the history of psychiatry's approach to sex and gender issues reveals that the values contained are often moral. He goes on to make the suggestion that, in general, psychiatric diagnosis would be improved if classifications that at present contain largely moral values could be replaced by non-moral values, 'adding a dimension of "sickness" to the dimension of "wrongfulness"' [7]. One advantage of the proposal is that it might reduce the amount of disagreement about constitutive values and thus add to clinical reliability.

This positive suggestion, albeit rather guarded, is an example of the kind of suggestion made in the final chapter for the future of the DSM. There, the key proposition is that values of clinical and administrative utility of DSM-IV should be replaced by the ultimate aim of aiding the mentally ill. Further, rather than concentrating narrowly on ill-health and a correlative notion of health it should focus explicitly on a broader notion of well-being or the Greek notion of eudaimonia. This would be to make the new DSM more explicitly evaluative and political. But, Sadler argues, it is better to make this explicit and thus subject to openly democratic control rather than letting it remain hidden and implicit.

Two features of Sadler's placement of values at the heart of his analysis of classification deserve further note. One is that, because it concerns the role of values in psychiatry, Sadler suggests at the start of *Values and Psychiatric Diagnosis *that it is merely the beginning of a conversation. The implication is that one should not expect to command agreement on such matters which call for political debate. (That said, Sadler himself opposes value-subjectivism and thinks that there is significant fact-like consensus about at least some values [8].)

Secondly, alongside discussion of aesthetic, ethical, pragmatic and epistemic values Sadler also discusses what he calls ontological values. These are connected to the assumptions about nature or reality and constrain the approach taken to psychiatric classification. Thus Sadler contrasts the biological naturalism that dominates psychiatry with other perspectives such as transpersonal, social constructionist and existential-phenomenological in order to reveal the assumptions in play. But here the centrality of values works least well. Had he instead treated these more directly as competing claims about reality that could be assessed for their truth he would have had sharper critical tools. By looking at them instead as primarily matters of value he makes assessment of their relative merits essentially indirect. It remains, however, an impressive demonstration of the role of applied philosophical analysis to the future development of psychiatric theory.

### Neil Pickering *The Metaphor of Mental Illness*

We suggested at the start of this review that analytic philosophy of psychiatry was reborn in the effort to come to grips with the status of mental illness. *The Metaphor of Mental Illness *[9] attempts to diagnose why there is continuing disagreement about the status of mental illness despite careful analysis. Neil Pickering argues that this is, in part, because of shared dependence on what he calls the 'likeness argument' which, he argues, is fundamentally flawed.

According to Pickering, the likeness argument is supposed to resolve the status of mental illness by showing that putative mental illness is, indeed, sufficiently like illness. It does this in one of two ways: either, it takes a paradigmatic form of illness, a specific case like hypertension or physical illness more generally, and shows that mental illness is sufficiently like it because it shares sufficient of its features; or, it abstracts a generic concept of illness (again, typically from physical illness) and shows that mental illness fits sufficient features of that general concept to count as illness.

Why, then, does the likeness argument fail to settle the matter? Pickering argues that it depends on two assumptions both of which can be questioned. He says:

If the likeness argument is to resolve this dispute two things must, I think, be taken to be the case:

1. that there are features of human conditions such as schizophrenia, which decide what category, or kind, these conditions area member of, and

2. that, with respect to the presence or absence of these features, a condition such as schizophrenia is describable independent of the category it is assigned to. [10]

The first is a general condition derived from a view of how concepts apply to things. The suggestion is that concepts apply in virtue of conditions having objective features. This stands in contrast, for example, to a view where all such concept application depends on an imaginative human judgement. The second is a more specific assumption relevant to the debate about mental illness. It is that the ascription of features to conditions – putative illnesses – can be made independently of a top down decision as to the illness-status of those conditions.

Pickering suggests that both assumptions can be questioned. But the more robust 'strong objection' turns on questioning the second assumption. His claim here is that ascription of features to conditions – putative illnesses – depends on the overall category – illness or not – into which they are placed. The argument for this is piecemeal. In each of three cases – alcoholism, attention deficit hyperactivity disorder (ADHD), schizophrenia – he offers competing descriptions of their basic features manifesting first an assumption that they are illnesses and second that they are not. The behavioural features of alcoholism, for example, can equally be described in terms of moral weakness or of causally determined pathological behaviour. The same data can be equally well interpreted in the light of opposing top-down theories. Pickering concludes: 'The detectable and observable features of alcoholism do not determine what description should be given of them' [11]. Arguing that this is a general feature of such contested cases, Pickering concludes that the features themselves cannot be used to determine to which overall category the condition belongs.

The second and third sections of the book develop an alternative account of mental illness based on an account of metaphor. The idea is that calling mental illness 'illness' is a metaphorical extension of the use of the word 'illness'. Pickering helpfully contrasts his own views with social constructionism and the 'strong programme' in sociology of knowledge. His criticism of the latter is that, whilst it claims that psychiatric categories *reproduce *social factors, the details of the analogy between the medical and the social do not allow an explanation or reduction of the medical in purely social terms. It does not debunk medical psychiatric distinctions described in their own terms. Similarly, social constructionism, properly understood, need not threaten the reality of psychiatric classifications. A sceptical conclusion about classification is not warranted by what is plausibly socially constructed.

The strong programme in the sociology of knowledge sought to show that mental illness was a reproduction of a social exclusion of some form. But it tripped up over the idea of reproduction. The sceptical social constructionist... seeks to show that ADHD is an individualisation of a social problem and so to unmask it. But it does not allow for the possibility that to individualise a problem might represent something other than a political environment or force. I have argued that in fact it represents a medical conceptualisation or force. [12]

The argument for the metaphorical status of mental illness proceeds by a kind of *via negativa*: dismissing alternative views that mental illness is a simile or secondary sense. Pickering also distances himself both from Szasz's suggestion that it is a metaphor and thus a deception of sorts and also from Champlin's criticism of Szasz. This last contrast is helpful in setting out the positive view. Pickering reports, favourably, Champlin's account of Szasz thus:

[Szasz] is saying that so-called mental illness and physical illness are completely different kinds of things linked only by metaphor... [13]

Champlin also agrees that 'Metaphor involves two things between which a gulf of difference lies such that it can be bridged only in the language' [14]. Thus Champlin's own attempt to show that mental illness is not a metaphor turns on deploying the 'logical continuities' [15] between mental and physical illness. But Pickering argues that Champlin's approach cannot work because, again, it is a likeness argument. The supposed continuities are not independent of a prior view that mental illness is indeed illness.

Pickering himself does not quite subscribe to the view of metaphor just summarised. He thinks that there may be continuities and yet the application of the classification still be a metaphor:

Metaphors are not utterly ruled out by logical continuities. If mental illness is a metaphor, there may still be continuities or likenesses. The point here is that if there are such continuities, they do not play a role in the assignment of kind contained in the metaphor. [16]

The problem with this account is that the idea of items being linked 'only by language' or of features not justifying the application of a term but being somehow constituted from it are very dark indeed and smack of a nominalist account of language which is neither spelled out nor justified in the book. (Talk of items being linked only by metaphor looks itself to be a metaphor!) But there is one further clue to what 'metaphor' might mean.

In a later example – the body as machine – Pickering argues that 'we cannot find facts about the body that will enable us to dispute whether or not it is a machine' [17]. The facts and the overall view 'come and go all together' [18]. This is again a statement of his critique of the likeness argument. So the precise model of metaphor which lies at the heart of his claim depends again on the likeness argument. Thus the heart of the book is the discussion of the likeness argument in the first half and it is worth questioning just what Pickering's diagnosis really achieves.

We are not sure that the criticism is as successful as Pickering suggests. His central claim is that detectable and observable features of a condition, a putative illness, cannot be described without begging the question of the pathological status of that condition. This is not, however, a surprising claim. If the correct description of the features is taken to imply a pathological status then, of course, it cannot be independent of the overall status. But even if it merely provides evidential support, Pickering has really only undermined a foundational version of the likeness argument. But most accounts of scientific theories now accept the theory dependence of data and an essential holism in theory testing. Most would reject a foundational approach to data, as Pickering himself later reports [19].

Rejecting foundationalism in favour of scientific holism does not show that the likeness argument (or some version of the likeness argument) cannot work as part of a broader investigation of illness. Nor does it amount to the stronger claim Pickering sometimes makes expressed in passages such as:

The causal and dysfunctional features of schizophrenia and alcoholism are *created *in the light of the kind of thing they are thought to be [20]

The relevant features of alcoholism *do not*, contrary to what it demands, *exist *independently of the category into which alcoholism is placed. [21]

These are radical constitutive claims that amount to a form of idealism about mental and behavioural features. The holism that Pickering highlights only establishes an epistemological point: that we cannot *establish *or *know *the nature of the features of a condition independently of establishing or knowing an overall classification. This is not the same as saying that the features are *constituted *as the features they are through human judgement.

So something akin to the likeness argument that Pickering protests against is still possible with the proviso that it is not thought of as a kind of foundational project. Judgements about mental and behavioural features and overall judgements about conditions form part of a larger package of ideas which have to be judged as a whole. This in turn threatens Pickering's account of metaphor unless he can find a way to unpack the distinction between items that are linked independently of linguistic categorisation and those whose connection depends on categorisation. If not, most of what he says is of a piece with the idea that in science, as elsewhere, observation and conceptualisation go hand in hand.

This is not, however, to play down the importance of Pickering's book. His careful articulation of shared assumptions by many working in the area and his critique of a foundational version of a central argument are very useful contributions to the literature as is the development of a novel alternative metaphorical approach. It will be interesting to see whether Pickering's is the last word on the status of mental illness.

### Giovanni Stanghellini *Disembodied Spirits and Deanimated Bodies*

Within the second area of our threefold division of recent philosophy of psychiatry, Giovanni Stanghellini's *Disembodied Spirits and Deanimated Bodies *[22] attempts to provide an account of what the experience of mental illness is like. Stanghellini works within a tradition of bringing phenomenological tools and analysis to bear in clinical findings that dates back to Jaspers. His book seamlessly weaves the two aspects together in order to provide an empirically grounded, but at the same time philosophically informed, account of core mental illnesses such as schizophrenia. But Stanghellini does not take an uncritical view of Jaspers. He argues that Jaspers' own approach to phenomenology is too intellectual, individualistic and depersonalised. Instead, drawing on ideas of Heidegger, Wittgenstein and Merleau-Ponty he argues that

[psychopathological] phenomena can only be gathered by interactive (emotional) involvement, not by dispassionate observation; concepts should not be used as labels of experience, but as expressions which function in an interpersonal context. [23]

Stanghellini suggests that the right path for psychopathology is suggested by two aspects of its history in the nineteenth century. Firstly, and negatively, he suggests that a future psychopathology should be contrasted with the move to place sufferers in asylums: a move, he implies, that was in part influenced by epistemological assumptions about the nature of madness. Subjects were spatially isolated in order to reveal the individualistic origins of illness. Secondly, he suggests that a seed for a better view of psychopathology can be found in early descriptions of the conditions labelled 'hebephrenia', 'catatonia' and 'heboidophrenia'.

Positively, these emphasised observable characteristic behaviour. Negatively, however, the descriptions rescinded away from the *significance *of behaviour and were described in almost mindless, behaviouristic terms. Thus Stanghellini commends as standing out the account of hebephrenia given by the psychiatrist Ewald Hecker in which patients' early life and experiences were described as a clue to their later state. This provides the germ of the idea defended in the book that 'schizophrenia is a pathology of the dialectical process between your own bare individuality and the mores and institutions to which you belong' [24].

The central substantive claim of the book is that schizophrenia is a breakdown of common sense. More precisely it involves a breakdown of three distinct areas: the ability to synthesize different senses into a coherent perspective on the world (coenesthia); the ability to share a common world view with other members of a community (sensus communis); and a basic pre-intellectual grasp of or attunement to social relations (attunement). Drawing on the work of Aristotle but also more recent phenomenologists Stanghellini says: 'The philosophical kernel of my proposal is to show how all these dimensions of the phenomenon of common sense (coenesthia, sensus communis, and attunement) are related to each other' [25]

The central chapters then outline how a description framed in philosophically rich language can be given of schizophrenia, as well as melancholy, manic depressive psychosis, verbal acoustic hallucinations and delusions. Psychopathological descriptions and philosophical accounts go hand in hand so that, for example, Aristotle's account of coenesthia sheds light on but is also illustrated by descriptions of schizophrenia. For example:

Schizophrenic persons experience a world in which sensory self-consciousness is disrupted. The sense of aliveness, the feeling of being embedded in oneself, the unity of self-experience is disrupted. This involves the experiencing of a dualistic Cartesian form of existence in which embodied self-consciousness is substituted... by incorporeal noetic self-consciousness.... [They] often describe their condition as that of a deanimated body or a disembodied spirit. Lack of sensory self-consciousness entails the feeling of being a lifeless body. [26]

Stanghellini describes his book as an essay: a piece of work firmly situated within a tradition but also critical of it and suggesting new developments. It comprises ten chapters or 'studies' and, although these are related, most are, more or less, independent pieces. Both the non-linear structure and the densely inter-textual style of writing makes this, at first, a difficult read. Some stages of the argument seem to go a little too quickly (such as the criticisms of 'behaviourism/functionalism', 'structural functionalism' and 'cognitivism'). Claims from Aristotle, Husserl or Merleau-Ponty are simply put forward without further initial justification or assessment. But in the main the idea is that the account as a whole stands or falls together. It is overall a hermeneutic project: if it gives a satisfactory theoretical description of both normal and abnormal psychology then that is an indication of its success.

There is, however, a tension at the heart of the project. One of Stanghellini's key aims is to understand mental illness according to descriptions given by sufferers themselves. But this is no easy task. Stanghellini himself offers a diagnosis of the difficulty:

Listening to a person affected by schizophrenia is a puzzling experience for more than one reason. If I let his words actualize in me the experiences he reports, instead of merely taking them as symptoms of an illness, the rock of certainties on which my life is based may be shaken in its most fundamental features. The sense of being *me *the one who is now seeing this sheet, reading these lines and turning this page; the experience of perceptual unity between my seeing this book, touching its cover and smelling the scent of freshly printed pages; the feeling that it is me the one who agrees or disagrees with what I am reading; the sense of belonging to a community of people, of being attuned to the others and involved in my actions and future; the taken-for-granted of all these doubtless features of everyday life, may be put at jeopardy. [27]

At the start of the book Stanghellini expresses some scepticism about Jaspers' claims about the 'ununderstandability' of psychopathology. But this remains a feature of the clinical encounter. The passage continues:

Although my efforts to understand, by suspending all clinical judgement, allow me to see these persons' self-reports as a possible configuration of human consciousness, I must admit that there is something incomprehensible and almost inhuman in these experiences, something that makes me feel radically different from the person I am listening to. [28]

This suggests, however, that the analysis offered might have a kind of 'as if' quality. The problem is that the explanatory framework of a breakdown of common sense presupposes the kind of (shared) intelligibility that, as Stanghellini reports, may be lacking. If it is, then it is not clear how much it makes sense to ascribe a breakdown of common sense or whether, by contrast, it serves as a kind of limit to understanding. The worry is that if common sense – in Stanghellini's specific sense – is a precondition for intelligibility then ascribing a breakdown of it cannot be part of an interpretative project. It would be, instead, an explanation from outside shared understanding.

In fact, Stanghellini seems to suggest that his aim is not so much a stable interpretation as a kind of piecemeal task which turns on shared emotional responses, as much as narrowly intellectual tools. In articulating some aspects of how such a task might go whilst at the same time explaining why it may prove so difficult, philosophical psychopathology faces its central challenge.

### Derek Bolton and Jonathan Hill *Mind, Meaning and Mental Disorder*

In the new version of *Mind, Meaning and Mental Disorder *[29], Derek Bolton and Jonathan Hill take up an historical dichotomy that was first brought to attention in psychiatry by Karl Jaspers in his *General Psychopathology*: the distinction between reasons, or meaningful connections, and causes, construed in terms of natural laws. An explanation of an action or behaviour by reasons looks very different from a causal account, and reason vocabulary seems to provide explanatory power that is not available through more basic causal resources. As a specific offshoot of the generic mind-body problem, the attempt to reconcile these two approaches to explanation has been the subject of much debate, with the tide of reductionist materialism taking meanings to be reducible to causal properties.

Bolton and Hill argue that meanings are causes but without attempting to reduce the former to the latter. This is achieved by redefining the boundaries of causation, abandoning the reason versus cause divide and replacing it with a distinction between intentional and non-intentional causation. Causation is intentional where behaviour, action or even more basic non-mental phenomena are best explained in terms of information carrying states. This incorporates much of the realm of biology in addition to psychology, thus shifting the boundary between the traditional 'natural sciences' and the domain of psychology. Moreover, intentional causation can be understood when couched in terms of function, as having its origin in biological systems that fulfilled the evolutionary purpose of survival. The main project of the book is devoted to setting out and justifying this thesis, before attempting to use the analysis to develop accounts of intentional explanation and mental disorder that render psychological phenomena explicable in the same way as biological events.

The authors' motivation for developing the intentional thesis arises from the undeniable predictive power of behavioural explanations given in terms of meaningful mental states, which is taken to indicate that mental states are causes of intentional behaviour. This premise is combined with the intuitive notion that the brain causally regulates action, and these two ideas are reconciled by the 'encoding thesis', which states that meaning is encoded in the brain. It is argued that this position allows meaningful mental states to be incorporated into a respectable science whilst simultaneously deferring to philosophical objections to reductive materialism and allowing that such states possess intentionality.

Given the distinction between intentional and non-intentional causation Bolton and Hill go on to describe how certain biological processes such as the regulation of blood pressure can be understood as instances of intentional causation. They do this to remove the connotation of 'mentality' from the idea of intentional causation and show that such an account can comfortably sit within a naturalistic framework and successfully explain phenomena that have traditionally been understood in terms of physical-causal processes. Locating the subject matter of psychology on a par with biology is intended to draw psychology closer to the realm of science whilst maintaining the insight that many psychological phenomena are irreducibly meaningful and necessarily involve interactions with the world.

The latter part of the book utilises the philosophical conclusions of the former part to argue for new interpretations of mental disorder as a whole, as well as focusing on several individual conditions specifically. The idea that biological systems possess intentionality opens up a normative dimension of explanation that does not arise in systems described at the purely physical-causal level. If a system can be assessed in terms of its functioning, it can be construed normatively, as either something that can function correctly or something that can dysfunction and go wrong.

A disorder arises when the intentional causal system is somehow disrupted, whether the cause is social, environmental, genetic or some other physical factors. The cause may be a non-intentional-causal factor or it may be a breakdown caused by competition within intentional causal systems.

In contrast to non-intentional causality the elements of intentional-causal processes do not necessarily work in harmony If we assume that the efficient function of an intentional-causal sequence in a physiological system has evolved over several million years, then the learning of new rules for perceptions and actions over hours, days, months, or even a few years, may seem to be a precarious truncation of the process! [30]

The authors use contemporary models of psychological disorder to support an analysis of several recognised conditions on an intentional-causal basis. They suggest that there is a complex interplay between intentional and non-intentional causal processes that biological psychiatry has historically overlooked, and outline several examples where their position may usefully serve to explain abnormal behaviour. The advantage of such accounts is that they can provide explanatory power irrespective of the supposed aetiology or causes of the dysfunction: thus we are not committed to 'explanations' of behaviour only being possible when physical-causal factors have been discovered.

This analysis serves as a model for philosophical investigation of psychopathology. But there remain some key difficulties with the book. One issue is how precisely the thesis that the brain encodes meaning should be understood. A second and related difficulty is an ambiguity about the concept of 'information' throughout *Mind, Meaning and Mental Disorder*. In places it appears to be used synonymously with 'content' or 'meaning' and thus helps defuse philosophical puzzles about how meaning is part of nature. But it can play that defusing role only because it is introduced in a much less philosophically charged way as, for example, what is encoded in patterns of neural transmission. It is now commonplace to use the concept of information in such sub-personal contexts. But this then raises the question of how information introduced in that manner can also be identified as information in the sense of mental content. What qualifies sub-personal information as personal level content is, presumably, the context in which the transmission occurs, i.e., in the brain of a subject situated within a worldly environment. But if so, the neuronal firing pattern itself (the state within the brain) cannot be said to *be *information in the sense of content. Furthermore, given the authors' claim to be taking a broadly Wittgensteinian view of meaning, it would be inconsistent if they took such firing patterns to be the *vehicles *of content since Wittgenstein, famously, rejects inner vehicles of content. So precisely what it is that is supposedly encoded in the brain remains unclear after lengthy discussion.

Bolton and Hill have attempted to re-evaluate the dominant Humean analysis of causation and show where psychiatry can progress on the basis of a new framework, in which the boundaries between reasons and causes are revised. Furthermore, they show how an analysis of mental disorder can be based on such a philosophical model. But it seems that more work is needed to clarify the heart of the new model.

### Pat Bracken and Phil Thomas *Postpsychiatry*

*Postpsychiatry *is perhaps best thought of as fitting the third aspect of our taxonomy. However it does not work within a conventional picture of the scientific status of psychiatry as it critically places assumptions about scientific psychiatry in a broad historical context.

In *Postpsychiatry *[31], Pat Bracken and Phil Thomas, psychiatrists who have trained in philosophy, aim to overturn several fundamental assumptions about the nature of mental health work and psychiatric practice and advocate a new approach to mental health that does not rely on narrowly medical definitions and treatments. Bracken and Thomas use the term 'postpsychiatry' to refer to the new approach, emphasising its position within an historical context as a progression beyond modernism. They set out their position in contrast to two deeply embedded aspects of the modernist assumptions found, they argue, in psychiatry: the primacy of science and its methodological focus on the individual.

Briefly tracing the development of ideas from Enlightenment thinking, Bracken and Thomas argue that faith in the ability of empirical science to yield progress and answers to questions about natural phenomena is a foundational assumption of academic psychiatry:

This continues to be the governing ideology of many academics: that the authority of psychiatry is based on its identification with science and technology [32].

Additionally, Enlightenment concerns with the nature of subjectivity led psychiatry to subscribe to a form of methodological individualism whereby disorders are understood as pathologies of mechanisms or processes 'inside' the individual. This is true as much for Jasperian phenomenology and psychoanalysis as it is for biological psychiatry. The authors are keen to highlight that these are not merely abstract academic issues. The Enlightenment focus on reason and the authority of medical expertise in dealing with madness have historically been tools of justification for using psychiatry as a means of social control, exclusion and coercion, and this is an issue at the heart of current debate about the role and nature of mental health services.

Bracken and Thomas emphasise that their position is not anti-science; rather, they are concerned to reflect on the limitations of modernist thinking and reject its claims to foundational, universally valid knowledge:

postmodern thought does not involve a *rejection *of reason, science or technology...postpsychiatry is about a realization that the...guiding assumptions of modernist psychiatry are only that: assumptions [33].

Following an initial outline of the policy background in the English NHS and the recent rise in user-led research, Bracken and Thomas move onto their central analysis. They discuss the 'gaze' of psychiatry, effectively guiding the reader through a brief history of the way Western psychiatry has perceived its subject matter. On the way, the views of Jaspers and Chomsky are briskly contrasted with Heidegger and the later Wittgenstein to demonstrate how different strands of Cartesian theoretical perspectives have shaped psychiatry's grasp of its subject matter.

Following this historical analysis, *Postpsychiatry *goes on to offer a positive analysis of the current state and possible future of psychiatric theory, policy and practice. They aim to dispute the modernist assumptions by challenging the presumed superiority of the scientistic approach to psychiatry, arguing that it should be tempered and set within a context where the methods and applications of science and technology are open to debate. Further they propose that mental illness can only be understood, explained and treated if social, cultural, familial, political and environmental factors are taken into account, rather than exclusively focusing on establishing empirically robust diagnoses and developing treatments on the basis of symptoms and diagnostic categories alone:

Postpsychiatry is our attempt to subdue the bright light of medical science: not because we want to get rid of it or to deny its benefits, but because we believe that the insights of other approaches are equally important and valuable. [34]

The framework of postpsychiatry is intended to provide a context in which clinical expertise is not only a matter of diagnostic judgement but also sensitivity to a multitude of priorities, values and pressures.

The mechanics of this project are set out in the second half of the book, with detailed discussions on such topics as first person narratives in illness and recovery, the implicit assumptions inherent in taking psychiatric case histories, and the implications of the proposed approach for policy and mental health in a global context. On the basis of their experience of psychiatric practice in the north of England, Bracken and Thomas detail some of the practical implementations of their revised approach to dealing with psychiatry and mental health that have successfully been put into place. Community groups as *Sharing Voices Bradford *place explicit emphasis on the centrality of the user and his/her needs and values for mental health service and care.

Contrary to the cynicism of much postmodern literature, Bracken and Thomas are cautiously optimistic about the prospects for postpsychiatry as a framework for implementing effective mental health care across different cultures and communities. They are keen to emphasise their distance from the pro/anti-psychiatry dispute arguing that the debate has moved into a new arena of discussion about best practice for dealing with mental distress. Psychopharmacology, for example, is not simply dismissed as an instance of scientism but should instead be discussed and offered in the context of an open, meaningful relationship between patient and professional.

Given the heavy philosophical and historical artillery used to set up the key tenets of postpsychiatry, the question naturally arises as to whether *Postpsychiatry *successfully hits its target. Crucially, the analysis of psychiatry is grounded in a critical analysis of the conceptual foundations of academic psychiatry. Much of the philosophical legwork is directed towards this critique, with the proposals of the postpsychiatry enterprise seemingly arising by default as the preferred approach once the limitations of the modernist approach, centred on control, individualism and the epistemic primacy of science, have been realised. Perhaps inevitably, in a book that covers such a broad range of topics within this interdisciplinary field, the relationship between philosophical argument and practical implications is at times tenuous, and it is not entirely clear precisely how the positive proposals for practice are justified or explained by the philosophical argument against modernity.

But philosophical and historical argument is not the only support that Bracken and Thomas appeal to. They argue that postpsychiatry is not only warranted in principle but is already beginning to have explicit positive effects, as evidenced by their own personal experience as psychiatrists working with community and service user groups in and around Bradford. These examples, rather more than the conceptual arguments, provide evidence for the efficacy of at least aspects of postpsychiatry in practice. The slogan 'Ethics before Technology', for example, represents a significant shift towards service-user centrality and the importance of cultural contexts in understanding mental health issues.

The strengths of *Postpsychiatry *do not lie in its attempt at historical and philosophical analysis so much as its outline of proposals for how psychiatric care should be developed. Its forward-thinking approach is backed up by a detailed though somewhat narrow sweep of the philosophy, history and development of psychiatry through a primarily humanistic critical lens, lending it an impressive philosophical artillery with which to support its main claims. It is a manifesto for a shift in thinking about mental illness that encourages multiple perspectives, opinions and voices to become visible and taken seriously in the ongoing effort to provide beneficial mental health care to those in distress.

The two final books considered here are edited collections.

### Julian Hughes, Stephen Louw and Steven Sabat *Dementia: Mind, Meaning and the Person*

Whilst the nature of the scientific and evaluative status of psychiatric diagnosis, or the meaning or content of bizarre delusions such as thought insertion, cry out for philosophical discussion, dementia, although a depressingly familiar condition, might be thought to be of less philosophical interest. But, as *Dementia: Mind, Meaning and the Person*, edited by the old age psychiatrist Julian Hughes, the geriatrician Stephen Louw and psychologist Steven Sabat, makes clear it strikes at the heart of our understanding of what it is to be a human subject [35].

By far the most common theme in this collection is how dementia threatens the status as a person of those who fall victim to it. But this prompts a further concern, reflected as a subsidiary theme of this collection: if the personhood of sufferers can be lost, will this not lead to a justification for abusive practice?

Most of the essays attempt to argue that personhood is not in fact undermined by dementia. We will consider a small proportion of the more central papers.

In a short paper, Harry Lesser distinguishes between two broad senses of 'personal identity'. One, corresponding to the main philosophical use of the phrase, is the sense of being the same individual person over time. The other, more common in psychology and sociology, is a person's conception of themselves. Focusing on the first, Lesser suggests that, whatever precisely (human) personal identity comprises, it involves the possibility of decline, including mental decline. 'The effects of dementia do damage the awareness of one's identity and can be particularly serious and troubling. But they give us no philosophical ground for saying that the identity has been destroyed, or that the relationship with them has been destroyed or should be ended...' [36]

Whilst this is a promising suggestion it does not explicitly address the arguments that dementia does undermine personhood because personhood is essentially tied to memory and memory is attacked by dementia.

John McMillan adds to Lesser's account a subtle variant. McMillan draws substantially the same distinction under the terms 'quantitative' versus 'qualitative' identity. Suggesting, with Lesser, that quantitative identity is not changed by dementia he argues that qualitative identity, which he connects to a notion of narrative, can be. Furthermore, he suggests, without further development, that sufficient change in narrative identity might indeed undermine duties and obligations by others to such a sufferer.

Jennifer Radden and Joan Fordyce develop the idea of narrative a little further. Again, adopting a distinction, drawn in this case from Ricoeur, between simple sameness ('idem') and a broader notion of self identity which involves a dialectic of self and others ('ipse' or 'ipseity') they argue that the latter has important connections to narrative. This enables them to go on to argue that identity in this second sense is the result of active collective authorship. Identity is 'a partly aspirational construct' [37]. There is a degree of selection in the construction of a meaningful self narrative. The process is active and social. This view has consequences for dementia.

Ricoeur's emphasis has been on the way the *formation *of a person's *ipse *identity depends on and is matched by the ipseity of those around them. But his account readily allows us to extend the 'bipolar' or reciprocal phenomenon to cover the *sustaining *of the dementia sufferer's *ipse *identity through the attention of others. [38]

There is, however, a central difficulty in assessing these claims. If the claim is as radical as Radden and Fordyce suggest then the construction involved cannot merely be one of social factors causally affecting individual personality. Everyone can agree that that is possible. Instead it needs to be a stronger claim – that personhood is constituted by social factors – but that requires much more explicit justification.

Social constructionism is itself is criticised by one essay in the collection (Thornton). In another, Michael Luntley provides a considered alternative constitutive account of personhood a consequence of which is that the self can itself be lost in severe dementia. Luntley's account is broadly Kantian. He argues that whilst Lockean theories of the self take for granted the identity of ideas or thoughts entertained by a subject they struggle then to give an account of the principle that gathers together a set of thoughts as belonging to a particular subject. (Narrative approaches might be thought to be neo-Lockean given Luntley's terminology.) One problem is that no attempt to take the self as something that can be infallibly tracked in thought seems promising given that most accounts of such tracking of objects, deriving, e.g., from Russell, presuppose the unity of the self that does the tracking.

Luntley argues, instead, that the self should be approached as that which makes the tracking of objects possible. The self is not tracked but is constituted partly by an ability to keep in contact over time with objects in the outer world. That clue in turn can then be unpacked by looking at the constitutive abilities for keeping track of objects. But it raises the following sort of threat to the unity of a self. A breakdown of sufficient severity of the ability to bind ideas together over time such as, for example, to keep track of objects in the world, indicates a loss of self. Notwithstanding such a subject's (if that is the right word) use of the pronoun 'I', without the underlying cognitive abilities, Luntley's account suggests that no self remains.

Whilst the majority of essays in this collection blend empirical, philosophical and ethical ideas together and aim to defend the view that selves continue to exist under the severe stress of dementia, it is worth indicating that it by no means follows that treating sufferers with dignity and respect requires that to be true. Dementia might indeed threaten the personal identity of a sufferer without justifying otherwise apparently abusive treatment.

### Bill Fulford, Katherine Morris, John Sadler and Giovanni Stanghellini *Nature and Narrative*

Finally, edited by the series editors Bill Fulford, Katherine Morris, John Sadler and Giovanni Stanghellini, *Nature and Narrative *[39], the launch volume of the series, is a collection of essays reflecting the variety of topics within the burgeoning domain of philosophy of psychiatry. The papers come from a wide range of theoretical and methodological perspectives, drawn together by the editors as examples of new ways of thinking about ethical and conceptual issues in psychiatry. With articles covering topics from the law to phenomenology, the sheer diversity of subject matter highlights the scope of this emerging field. Despite their diversity, all the papers point towards a rejection of the explanatory authority of reductionist materialism that has recently pervaded the theory and practice of psychiatry. Such reductionism is evidenced by the recent predominance of psychopharmacology: its biochemical method of action has been perceived not merely as one possible approach to the treatment of psychiatric symptoms, but as the fundamental root of psychiatric disorders.

Although the book is explicitly conceived as the first book in the series and hence in one sense introduces the new philosophy of psychiatry, it does not contain introductory or more basic material so much as serve as a launch pad for other texts in the series, to engage the attention of a wide readership from a multiplicity of backgrounds, lay and academic alike.

Inevitably, in an interdisciplinary book that is designed to appeal to a wide audience, the philosophical argument is not always as careful and painstaking as one would expect from a single topic philosophical text. Similarly, descriptions of psychopathological phenomena are at times abbreviated. Nonetheless the collection contains a number of articles with particularly original ideas or insight, or deploying established ideas to broach new conceptual terrain in psychiatry, from the perspectives of philosophy, ethics, psychology, phenomenology and science.

In one of his last papers, the late Wittgenstein-scholar Gordon Baker explores Wittgenstein's project of providing 'therapy, not theory' by dissolving apparent philosophical problems through clearer understanding of the concepts involved, drawing parallels with the nature of psychotherapy. In another chapter, the phenomenologist Eric Matthews uses Merleau-Ponty's notion of embodied consciousness to steer between biological reductionism and an anti-psychiatric dismissal of the reality of mental illness, in an article that perhaps best summarizes the new direction of thought in the field.

Discursive and constructionist approaches to mental disorder are considered by both Rom Harré and Grant Gillett, again with the emphasis placed on the inadequacy of the medical model and physical-causal concepts in explaining and understanding disorder. The focus is less on articulating a precise, positive account of the discursive approach than on criticism of opposing positions. Phenomenological perspectives on body dysmorphic disorder and schizophrenia are delivered by Morris and Depraz respectively, providing specific examples of how the application of phenomenological arguments can help account for and explain behaviours and actions that seem incomprehensible from a strictly medical standpoint.

Heinimaa's paper on the un-understandability of some aspects of psychiatric disorders addresses a central issue in the philosophy of psychiatry, querying whether the words and behaviour of mentally ill individuals are essentially meaningless, or have an underlying meaning that is simply not accessible from a viewpoint of one not afflicted by the illness. This has implications for philosophical discussions on the normativity of meaning and the determination of mental content, but also direct practical implications for the ability of a professional or clinician to understand a patient and establish a meaningful therapeutic relationship. Such issues are of key importance to psychiatry, and Heinimaa's discussion will hopefully provide a platform for further debate in the area.

More so than other books so far published in the series, *Nature and Narrative *is an explicit attempt to introduce a broad spectrum of readers to philosophical concepts and issues underpinning mental health care. Whilst this means that at times things go rather quickly, it gives a sense of the developing field that will hopefully attract and intrigue potential readers.

## The future of philosophy of psychiatry

If the *International Perspectives in Philosophy and Psychiatry *represents some of the themes making up the present state of philosophy of psychiatry, what of the future?

One possibility is that the diversity of the new philosophy of psychiatry is a temporary phenomenon. It might be, in Kuhnian terms, an aspect of the revolutionary birth (or rebirth) of the subject within broader analytic or Anglo-American philosophy. If so, one might expect it to evolve into its normal science phase, characterised by an agreed agenda of puzzles to be worked through within an agreed methodological framework. It might become more like the philosophy of mind or epistemology with a settled role within the philosophical canon.

However, we do not believe that the subject will or should evolve like this. Philosophy of psychiatry, or more broadly, philosophy of mental health care is primarily a philosophy of and for mental health care. It is at its best when it responds to questions and examines the conceptual underpinning of developing thought in this area. This in turn makes prediction of the future of philosophy of psychiatry difficult.

But, if we are to engage in a little 'futurology', it seems reasonable to expect that, in the short to middle term, there will be development in response to concerns currently arising in broader backgrounds of mental health care and philosophy. To pick a few areas in which we expect to see further development:

From the practice side, we expect a growth of emphasis on the role of the individual in diagnosis and assessment, in line with increasing dissatisfaction with a reductionist medical model of mental illness. Furthermore, the development of models of comprehensive diagnosis and assessment by the WPA will prompt discussion of how individuality or subjectivity is best captured, possibly in narrative or other terms. However, the notion of an individual-based or, perhaps, ideographic (by contrast with nomothetic) component in comprehensive diagnosis is potentially in conflict with a second change in emphasis expressed by the APA: the need for the next revision of the DSM to possess validity as well as reliability. Conventional, nomothetic models of validity have typically emphasised the need to prescind away from individual 'surface' details to capture underlying structures, and this is in tension with the recent emphasis on the need to capture precisely the surface details that comprise a subject's experiences and go to make up a meaningful life as whole. The task of resolving this tension will come to the forefront of discussions of psychiatric taxonomy and diagnosis as new editions of the DSM and ICD are developed.

There has been a longstanding aim on the practice side to develop models of mental health recovery which are not merely negatively characterised as the successful treatment of illness. Whilst some progress has been made by the 'recovery movement' there has been little philosophical input to help to articulate a genuine alternative understanding as to recovery might comprise and how it connects to empowerment, the avoidance of stigma and other key concepts. We expect work on the 'logical geography' of these concepts that will contribute to how the idea of recovery is understood.

From the philosophy side, the recent growth of embodied, embedded and enactive approaches to characterising human experience within philosophy of mind promise also to shed light on psychopathological experience. We expect that enactivism from the broadly analytic philosophical stable will be used to augment more traditional continental philosophical resources to characterise the phenomenology of mental health and illness. Additionally, we also expect it to be explored as a resource to contrast with representationalist approaches for interpreting data from brain imaging experiments, particularly where structural or functional differences in such brain imaging data are touted as evidence for the aetiology of specific psychopathologies.

Another development from the philosophy side is a resurgence within moral philosophy of virtue ethics. This emphasis on the nature of the moral agent has so far been extended, within philosophy, to epistemology, in so called virtue-epistemology. But it promises also to shed light on the nature of expertise involved in clinical judgement -judgement of both facts and of values – and to suggest the limits to what can be explicitly codified.

Within the UK there are currently two issues high on the health care policy agenda, on which research in philosophy of psychiatry could potentially influence. These concern human rights and risk analysis.

The IPPP edited collection on dementia illustrates well how the human rights of sufferers could be ignored, owing to a denial of the status of personhood to a patient; a difficulty that is relevant across the field of mental health where the rationality of a patient is in question. Related concerns regarding autonomy, coercive treatment and the deprivation of liberty on account of potential risk, although typically considered to be predominantly ethical issues, are highly contentious areas of debate in which we consider a clearer understanding of the meaning of subjects' experiences would be invaluable. A more explicit acknowledgement of the range of societal values in play in such assessments may additionally contribute to the formation and implementation of future mental health policy.

Overall it seems then that we live in interesting philosophical times, in which there is potential for a fruitful crossover between the disciplines of philosophy, psychiatry and mental health care that may generate a genuine and beneficial impact on mental health practice. We look forward to the next stage of the development of the philosophy of mental health, both for the conceptual challenges that will be posed and the longer term differences it could make to mental health care.
